# Supplementary material for: Development of a computer-aided design software for the quantitative evaluation of aesthetic damage
Source: PLoS One. 2019 Dec 18;14(12):e0226322. doi: 10.1371/journal.pone.0226322 (PMC6919621; doi:10.1371/journal.pone.0226322)
Supplement: S1 Code site — 1. (ZIP) [file pone.0226322.s001.zip › [EESC_jr][Projeto]Relatorio_07_21/vendors/select2/docs/_includes/examples/disabled-results.html]

# Disabled results

Select2 will correctly handle disabled results, both with data coming
from a standard select (when the `disabled` attribute is set)
and from remote sources, where the object has
`disabled: true` set.

First
Second (disabled)
Third

{% highlight html linenos %}
First
Second (disabled)
Third
{% endhighlight %}
